# Development and Optimization of Retinyl Palmitate-Loaded Oleogel and Nanoemulsion Designed with Potential for Microneedling Treatment

**DOI:** 10.3390/ph19081247

**Published:** 2026-08-08

**Authors:** Aleksandra Tobiasz, Agnieszka Gawin-Mikołajewicz, Katarzyna Wach, Alina Jankowska-Konsur, Bożena Karolewicz, Danuta Nowicka

**Affiliations:** 1 University Centre of General Dermatology and Oncodermatology, Faculty of Medicine, Wroclaw Medical University, 50-556 Wroclaw, Poland; alina.jankowska-konsur@umw.edu.pl (A.J.-K.); danuta.nowicka@umw.edu.pl (D.N.); 2 Department of Drug Form Technology, Faculty of Pharmaceutical Sciences, Wroclaw Medical University, 50-556 Wroclaw, Polandbozena.karolewicz@umw.edu.pl (B.K.)

**Keywords:** retinyl palmitate, microneedling, oleogel, nanoemulsion, dermatology formulation

## Abstract

**Background/Objectives:** The development of novel formulations that could have beneficial properties combined with dermatological procedures such as microneedling remains a challenge. The group of patients that could benefit from such a novel protocol is large and includes patients with acne scars. Both topical retinyl palmitate and the microneedling procedure have strong fibroblast-stimulating properties. **Methods:** In the study 5% *w*/*w* retinyl palmitate-loaded nanoemulsions and oleogel were prepared and examined to obtain formulations that meet criteria eligible for formulations combined with microneedling. All formulations were designed for aseptic preparation. Multiple nanoemulsion compositions were tested to select the optimal one. The selected 5% *w*/*w* retinyl palmitate-loaded nanoemulsion and oleogel were tested. **Results:** While retinyl palmitate-loaded oleogel met proper pharmacopeial norms for drug content and stability, the retinyl palmitate-loaded nanoemulsion, even though it demonstrated adequate physical parameters, maintained active substance losses during the manufacturing process above required limits. **Conclusions:** The retinyl palmitate-loaded nanoemulsion and oleogel remain promising formulations that could potentially be combined with microneedling. Considering its high sensitivity to manufacturing conditions, preparation of the retinyl palmitate-loaded nanoemulsion still remains a challenge.

## 1. Introduction

Acne scarring remains a common aftereffect of acne vulgaris, which significantly affects patients’ quality of life. The large patient population affected by acne scarring of different severity necessitates the development of effective therapeutic regimens [1,2]. In dermatology, the continuous advancement of innovative procedures, formulations, and treatment protocols remains a key area of research and development [3,4]. Effective acne scar treatment usually requires a multimodal approach, involving the synergistic effect of modern technologies and therapeutic agents. One of the methods frequently used in acne scar treatment is microneedling, already a widely adopted procedure in esthetic medicine. This method is commonly implemented for skin rejuvenation, with its potential to stimulate collagen and elastin production. Nevertheless, it has also shown great potential in the treatment of many dermatological conditions [5,6].

The mechanism behind microneedling is the process of creating multiple tiny channels of different depths in the skin, which induces a cascade of wound-healing processes. Such injury triggers processes of growth factor release, fibroblast stimulation, and finally tissue remodeling caused by synthesized collagen, elastin, and glycosaminoglycans [7]. To date, many different devices have been developed to perform such procedures—including manual dermarollers [8] and automatic devices, such as Dermapen^®^, which enable the adjustment of procedure parameters. Additionally, automatic devices minimize skin trauma compared to manual techniques [9,10].

Needling the skin not only stimulates its regenerative potential but also enhances active substance penetration. To potentiate the process of scar tissue remodeling, incorporating an active substance into the method seems to be an optimal solution [11]. Vitamin A (retinyl palmitate) and its derivatives have a well-established position in dermatology with great potential to encourage reorganization of scar tissue. Vitamin A and retinoids through nuclear retinoic acid receptors (RAR-alpha, beta, gamma) and retinoid X receptors (RXR-alpha, beta, and gamma) affect gene transcription, which leads to regulation of keratinocyte differentiation and proliferation [12]. Additionally, these substances exhibit anti-inflammatory properties, enhance expression of genes for collagen I and III in fibroblasts, affect the ECM (extracellular matrix), and decrease expression of matrix metalloproteinases, enzymes that cause collagen degradation [13]. Despite its broad range of biological activities, vitamin A (vit. A) remains very susceptible to external factors such as high temperature, ultraviolet (UV) radiation and oxidation, which causes significant difficulty in designing formulations containing it [14].

To date, the effectiveness of the microneedling procedure and its various combinations for acne scars have been well documented [15]. The most extensively described approaches include the combination of microneedling with chemical peels [16] and autologous platelet-rich plasma (PRP) [17]. The limited available evidence, including the pilot study by Zonunsanga, evaluates retinoids as part of a multimodal therapeutic protocol involving glycolic acid peels, microneedling, topical tretinoin, and salicylic acid peeling, rather than as an independent adjunct to microneedling therapy [18].

In contemporary research, a wide range of advanced pharmaceutical carriers for cutaneous delivery, including nanotechnology-based systems such as nanoemulsions and nanoemulgels, have been developed to enhance drug delivery performance [19]. Among modern carriers, oleogels have emerged as promising semisolid vehicles and are increasingly integrated into pharmaceutical practice. An oleogel is a type of semisolid, hydrophobic base, which makes it possible to design simple and innovative multiphase formulations. It has superior application properties compared to classic hydrophobic bases, like Vaseline. It combines certain occlusive properties with lighter texture [20,21]. Due to the absence of water, oleogel-based formulations exceed potentially good microbial stability [22]. A novel type of formulation emerging in the pharmaceutical and cosmetic industry for microneedling is nanoemulsions. They are a dispersed, liquid system where droplet size ranges between 20 and 200 nm. Like in classic emulsions, nanoemulsions are also divided into oil-in-water (o/w) and water-in-oil (w/o) ones. Preferable to classic emulsions, they have better absorption of the active substance profile and bioavailability, better stability, and they make it possible to incorporate hydrophobic substances. Finally, an undeniable advantage is their consistency and good application properties [23,24].

Nanoemulsions can be manufactured using high-energy (high-pressure homogenization—HPH, microfluidization, ultrasonication) and low-energy methods (phase inversion emulsification method, self-nanoemulsification method). While low-energy methods are suitable for heat-sensitive substances, they require the use of greater amounts of surfactants, produce less stable formulations and are less efficient than high-energy ones [25]. In contrast, high-energy methods, especially HPH, offer several advantages for both laboratory- and industrial-scale production, including process scalability, ease of operation, reproducibility, high throughput, and short processing times [26].

These two distinct drug delivery systems were selected because of their individual features. In a nanoemulsion, due to the small droplet size, the incorporated lypophylic substance can be effectively dispersed whereas in oleogel-based formulations, the structured lipid matrix enhances stability, provides a certain degree of protection for the active substance, and has a favorable texture [20,21,22,23,24].

The objective of this study was to develop retinyl palmitate-loaded oleogel and nanoemulsion formulations suitable for combination with microneedling procedures in patients affected by acne scarring. The formulations were prepared under sterile conditions, with optimized composition, minimizing the concentration of surfactants with optimal physicochemical stability.

## 2. Results and Discussion

### 2.1. Preparation of Oleogel with 5% w/w Retinyl Palmitate

The 5% *w*/*w* retinyl palmitate oleogel was manufactured using an Unguator^®^ (mixing level 1, 30 s) in a process that resulted in a significant reduction in active substance content to 88.43 ± 0.62% of the declared value, below the pharmacopeial acceptance range of 90–110% [27]. Consequently, an alternative method involving manual mixing with a mortar and pestle was selected, which resulted in retinyl palmitate content of 100.27 ± 0.69% of the declared value.

As a result of the described processes, a yellowish semisolid formulation was obtained. Photographs of the formulations prepared using both methods are provided in the Supplementary Materials.

Retinyl palmitate is known to be highly sensitive to external factors such as heat and UV radiation [28]. Even a minor heat level, such as that produced during the 30 s mixing process using an automatic machine, could cause significant losses. A study by Temova Rakuša et al. demonstrated that increasing the storage temperature from 25 °C to 40 °C leads to a significant reduction in retinoid content, commonly used in cosmetic formulations [29].

Since the formulation was intended for use during microneedling procedures, which involve the application of the formulation on the disrupted skin surface, the selection of oleogel as a base was considered appropriate. Several studies have described the use of oleogel-based formulations in patients with an interrupted skin barrier. Schwieger-Briel et al. studied a betulin-based oleogel in a group of epidermolysis bullosa patients. In such cases, where the appearance of wounds is constant, the selection of a proper non-irritant base is of great importance. The study showed promising results regarding its non-irritant and healing properties, which suggest oleogel could be an optimal base for formulations used on the disrupted skin barrier [30].

#### 2.1.1. Oleogel Viscosity and Spreadability Determination

Viscosity is a formulation’s resistance to flow on the surface to which it is applied. The viscosity of the obtained oleogel at a shear rate of 192 s^−1^ was 304.12 ± 10.47 mPa·s at 25 ± 1.0 °C and decreased to 165.35 ± 4.89 mPa·s at 36.0 ± 1.0 °C.

A temperature of 25 °C represents room temperature, whereas a temperature of 36 °C was selected in order to approximate the temperature within the dermis, as the formulations were expected to be distributed during the microneedling procedure on the disrupted skin barrier [31].

A lowering of viscosity at higher temperatures was observed. This is a typical phenomenon—semisolid formulations become runnier at higher temperatures in contact with the skin [32]. Considering the use of the designed oleogel in combination with microneedling, the measured parameters seem to be sufficient to formulate a product that does not flow from the surface of the skin too easily, and at the same time, is not too runny during application with a syringe.

**Figure 1:**
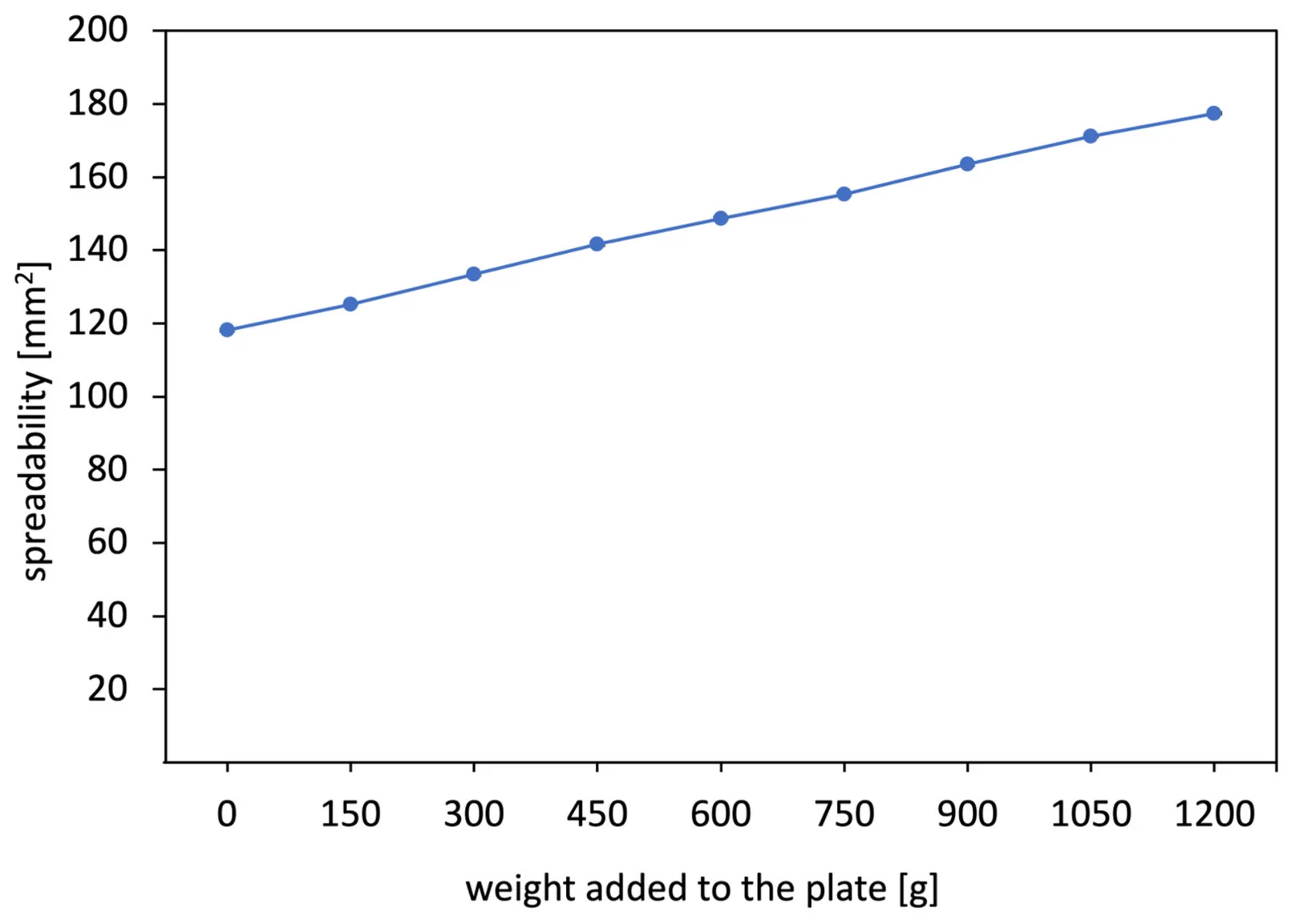
Spreadability of 5% *w*/*w* retinyl palmitate oleogel.

Spreadability is another important parameter characterizing semisolid formulations and reflects the ease with which a formulation can be distributed over the surface to which it is applied. As shown in Figure 1, the spreadability of the 5% *w*/*w* retinyl palmitate-loaded oleogel increased proportionally with the applied load. The spread area increased from approximately 118.12 ± 0.32 mm^2^ without additional weight to approximately 177.41 ± 0.28 mm^2^ at the maximum applied load of 1200 g. The formulation exhibited good spreadability even at low weight, whereas further load resulted in gradual and relatively small increases in the measured diameter. The obtained results indicate that the examined formulation can be spread on the skin evenly and effortlessly, which is desirable when combining the formulation with microneedling procedures. During microneedling, the formulation is spread on the surface of the skin using a microneedling device. Therefore, adequate spreadability is essential to ensure homogeneous application and continuous coverage of the treated area.

The optimal formulations’ viscosity and spreadability also determine proper adhesion and increased absorption of the active substance. In the study of Smeu et al., betulinic acid-loaded oleogel consisting of Compritol 888 ATO and an 85% *w*/*w* oil phase (a mixture composed of sunflower oil and olive oil in a 2:1 ratio) was examined and exceeded comparable viscosity at 25 ± 2 °C in the studied formulation [33]. Furthermore, the oleogel forms a thin occlusive layer on the skin’s surface, which may improve patient comfort during the procedure. A thin layer of product, which remains on the surface of the skin during the procedure, makes the procedure less painful for the patient. Such a phenomenon of pain reduction after covering nerve endings with an occlusive layer has been described in studies regarding wound healing [34] and is an important element of patients’ comfort during the procedure.

#### 2.1.2. Drug Content and Physicochemical Stability of Oleogel

The 5% *w*/*w* retinyl palmitate-loaded oleogel samples were stored under refrigerated conditions (6 ± 0.5 °C) in syringes protected from light with aluminum foil in order to minimize UV-induced degradation. A gradual decrease from approximately 100.27 ± 0.69% (day 0) to 94.96 ± 0.02% (day 28) in retinyl palmitate content was observed during storage. Nevertheless, after 28 days, the active substance concentration remained within the pharmacopeial acceptance range (>90%) [27].

Additionally, the examined formulations did not exhibit any macroscopic changes over time. The obtained results indicate that an oleogel base for retinyl palmitate seems to be a satisfactory choice. Oleogels have previously been described as stable and effective carriers for the delivery of various bioactive compounds [35], which supports their suitability as a vehicle for retinoid-containing topical formulations.

### 2.2. Optimization of 5% w/w Retinyl Palmitate-Loaded Nanoemulsion Preparation

The optimization process of retinyl palmitate-loaded nanoemulsions was focused on obtaining formulations characterized by appropriate physicochemical stability, small particle size, narrow particle size distribution, and low surfactant concentration suitable for application on the disrupted skin barrier during microneedling procedures. Nanoemulsions were prepared using the high-pressure homogenization (HPH) method, which is recognized as an effective and reproducible high-energy technique suitable for obtaining nanosized droplets with favorable stability profiles.

Tween 20 and Kolliphor EL were selected as non-ionic surfactants because of their relatively low toxicity and reduced irritation potential compared to ionic surfactants. Triacetin was incorporated as a cosurfactant to improve the stabilization of the dispersed phase while minimizing the total concentration of surfactants within the formulations [36,37,38,39,40].

Tween 20 and Kolliphor EL lower the interfacial tension, enabling the formation of smaller droplets. They also enhance the formulations’ stability by creating a protective layer at the oil/water interface. Higher concentrations of surfactants mostly enable the creation of more dispersed colloidal systems. Such an effect is only observed in a certain surfactant concentration range, above which micelle formation occurs, and droplet size reduction is limited [41].

In the study by Algahtani et al., surfactants generally recognized as safe, including Tween 20, Kolliphor, and Transcutol, were used for the development of retinyl palmitate-loaded nanoemulgels. The obtained formulations demonstrated favorable physicochemical characteristics and satisfactory stability. However, compared to the formulations developed in the present study, those nanoemulgels contained substantially higher concentrations of surfactants, which may increase the risk of skin irritation, particularly when applied on a disrupted skin barrier following microneedling procedures [42]. Additionally, the study by Abdollahi et al. mentions that non-ionic surfactants (Tween 20, Kolliphor EL) have less irritant potential compared to ionic surfactants. At the same time, the fact that a higher concentration of surfactants leads to higher irritation risk, even in the case of non-ionic surfactants, should be remembered [43]. Considering the necessity of maintaining relatively low surfactant concentrations while achieving adequate nanoemulsion stability, the incorporation of a cosurfactant was considered an optimal approach. To achieve it, Triacetin was selected. Similarly, in the study of Radomska et al., the addition of Triacetin to a microemulsion containing retinyl palmitate resulted in a stable formulation [44]. The performed evaluation demonstrated that the development of a stable retinyl palmitate-loaded nanoemulsion using minimal concentrations of surfactants and auxiliary substances, while avoiding the addition of preservatives, remains challenging. Due to the highly lipophilic nature of retinyl palmitate, achieving satisfactory physicochemical stability with reduced surfactant and cosurfactant content required careful optimization of the formulation’s composition and manufacturing conditions.

#### 2.2.1. Measurement of Particle Size (PS) and Polydispersity Index (PDI)

**Figure 2:**
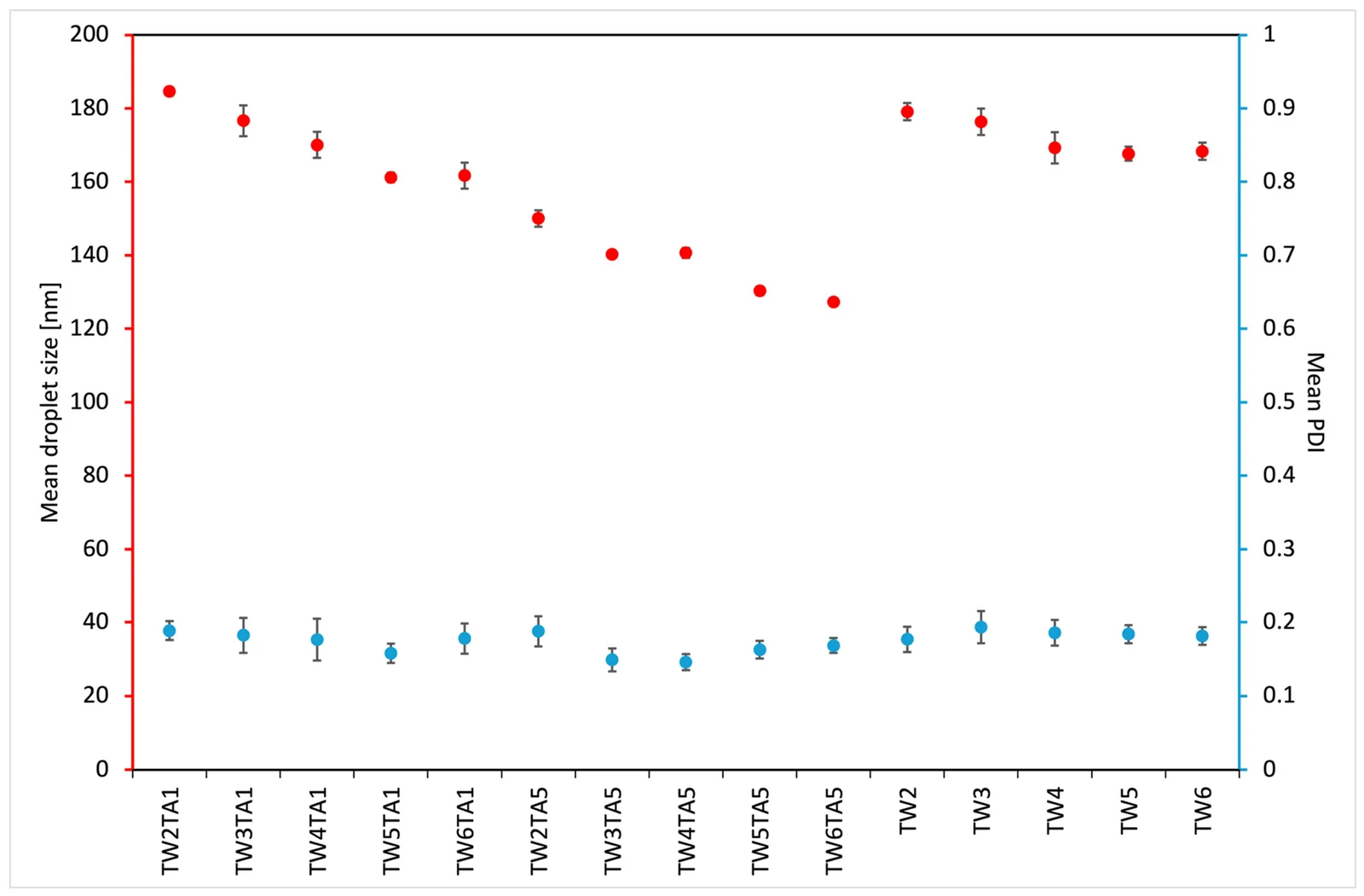
The mean droplet diameter (red marker) and PDI (blue marker) of the prepared 5% *w*/*w* retinyl palmitate-loaded nanoemulsions containing Tween 20 as surfactant and Triacetin as cosurfactant.

**Figure 3:**
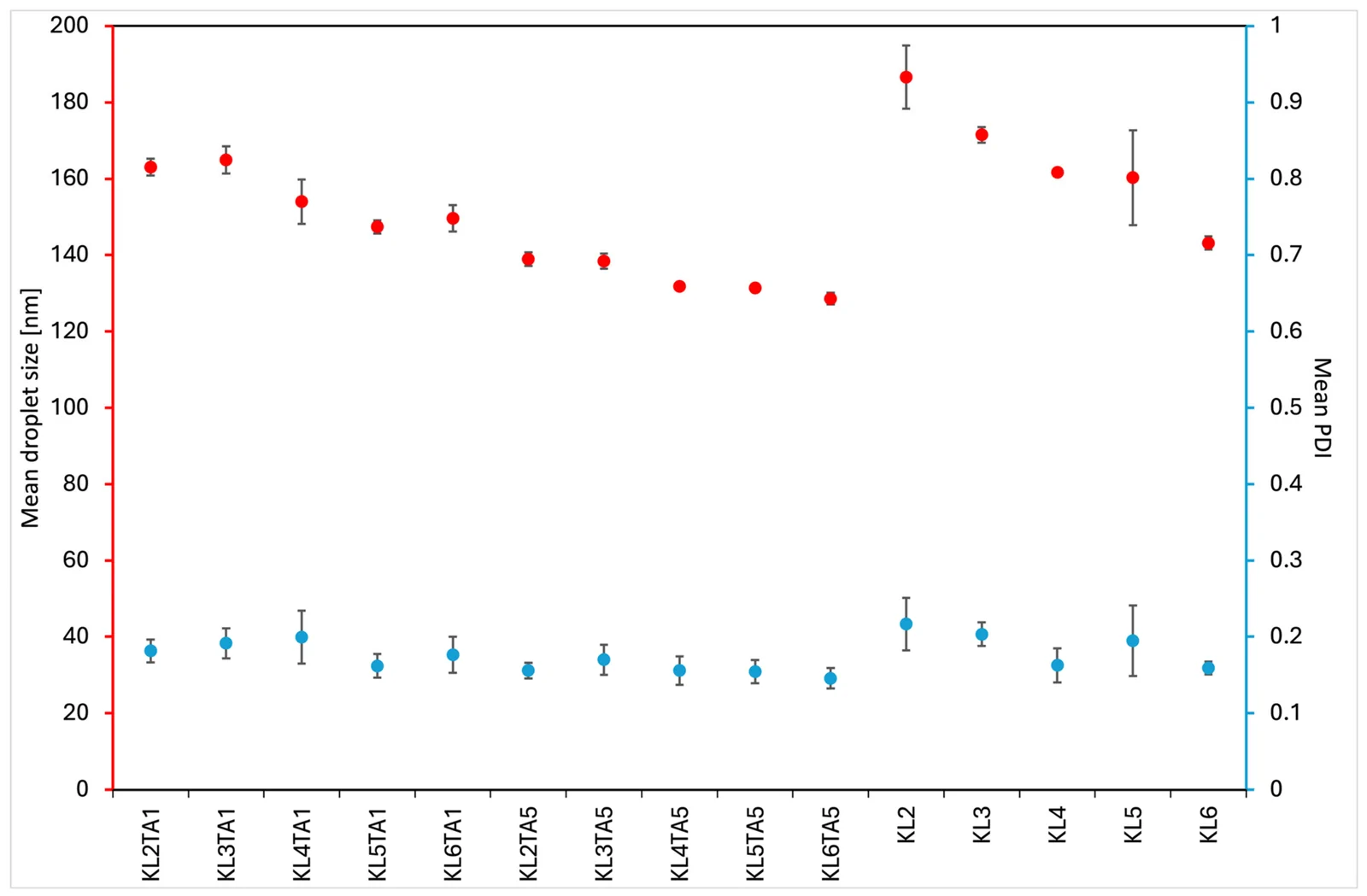
The mean droplet diameter (red marker) and PDI (blue marker) of the prepared 5% *w*/*w* retinyl palmitate-loaded nanoemulsions containing Kolliphor EL as surfactant and Triacetin as cosurfactant.

To evaluate the effectiveness of the selected emulsifiers (TW, KL), as well as the cosurfactant (TA), in stabilizing and encapsulating the oil phase within the nanoemulsion system, a series of formulations with varying excipient concentrations was prepared. The concentrations of surfactants were tested within the range of 2–6% *w*/*w*, while Triacetin was incorporated at concentrations of 1% or 5% *w*/*w*. The formulations were subsequently characterized by measurements of PS, PDI, and ZP (zeta potential) to identify the compositions with the most favorable physicochemical properties. The performed analysis demonstrated that surfactant concentration had a substantial influence on the average particle size of the developed nanoemulsions (Figure 2 and Figure 3). An increase in surfactant concentration resulted in a reduction in particle diameter. Furthermore, in this study, it was observed that a higher concentration of TA (5%) as a cosurfactant significantly decreases the particle size in the dispersed phase.

Currently, according to the literature, nanoemulsions are characterized by mean particle sizes below 200 nm [45]. All developed formulations exhibited particle sizes below 200 nm and PDI values lower than 0.25, which may indicate good homogeneity and physicochemical stability in the nanoemulsion systems. Following composition optimization, the most optimal formulations were identified as those containing KL and TW (5%, 6%) as surfactants, together with TA (5%) as a cosurfactant. These formulations were characterized by the smallest average particle size in the dispersed phase, in the range of 127.24–131.44 nm, and PDI in the range of 0.146 to 0.168 (Table 1), indicating good homogeneity and narrow particle size distribution.

#### 2.2.2. Zeta Potential Determination

Zeta potential is a key parameter used to evaluate the surface charge of particles and the electrostatic repulsion between similarly charged droplets within the dispersed phase. Higher absolute zeta potential values, whether positive or negative, are generally associated with enhanced physical stability, as they reduce the likelihood of particle aggregation, flocculation, and coalescence [46].

**Figure 4:**
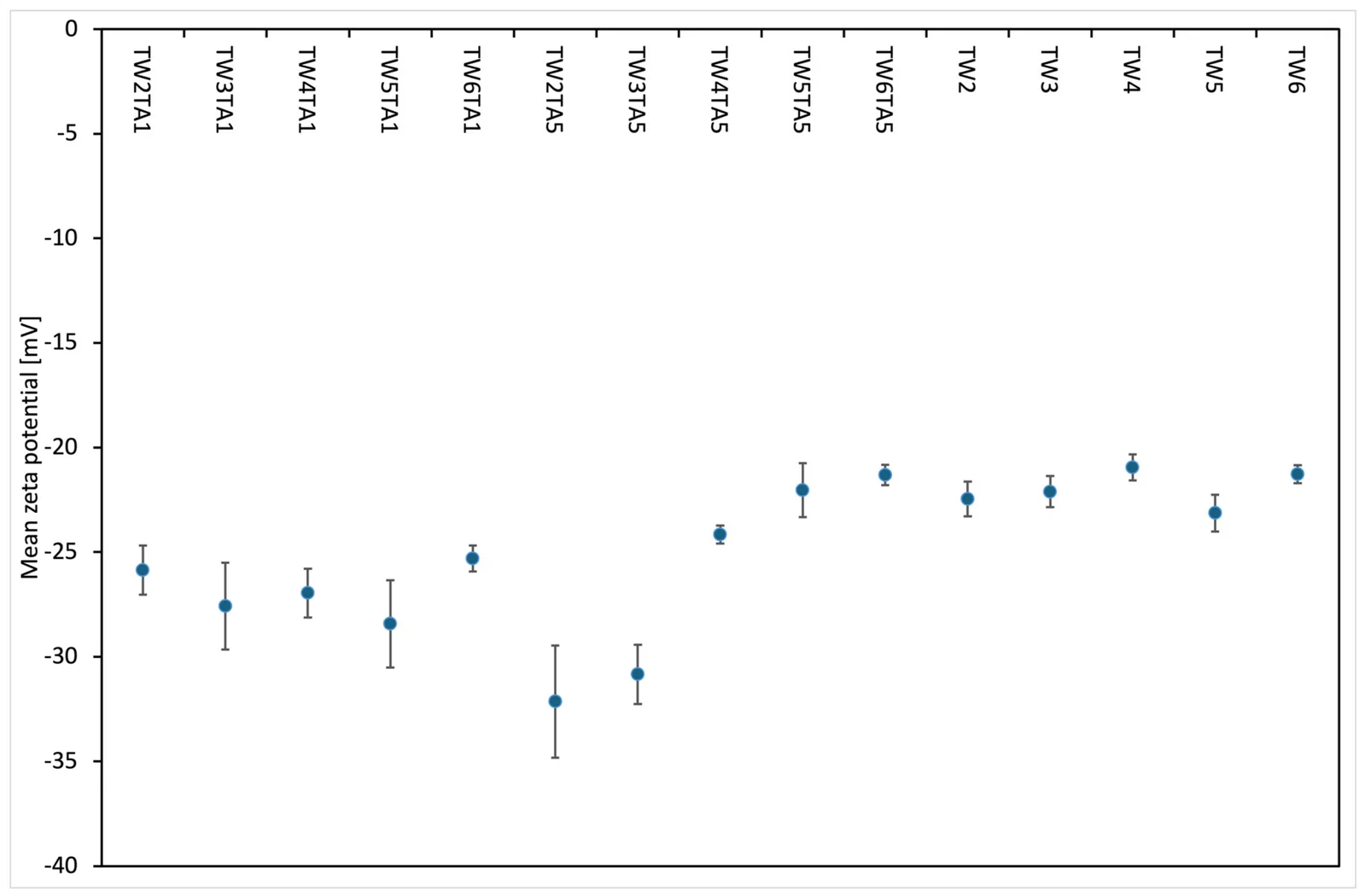
Zeta potential of the prepared 5% *w*/*w* retinyl palmitate-loaded nanoemulsions containing Tween 20 as surfactant and Triacetin as cosurfactant.

**Figure 5:**
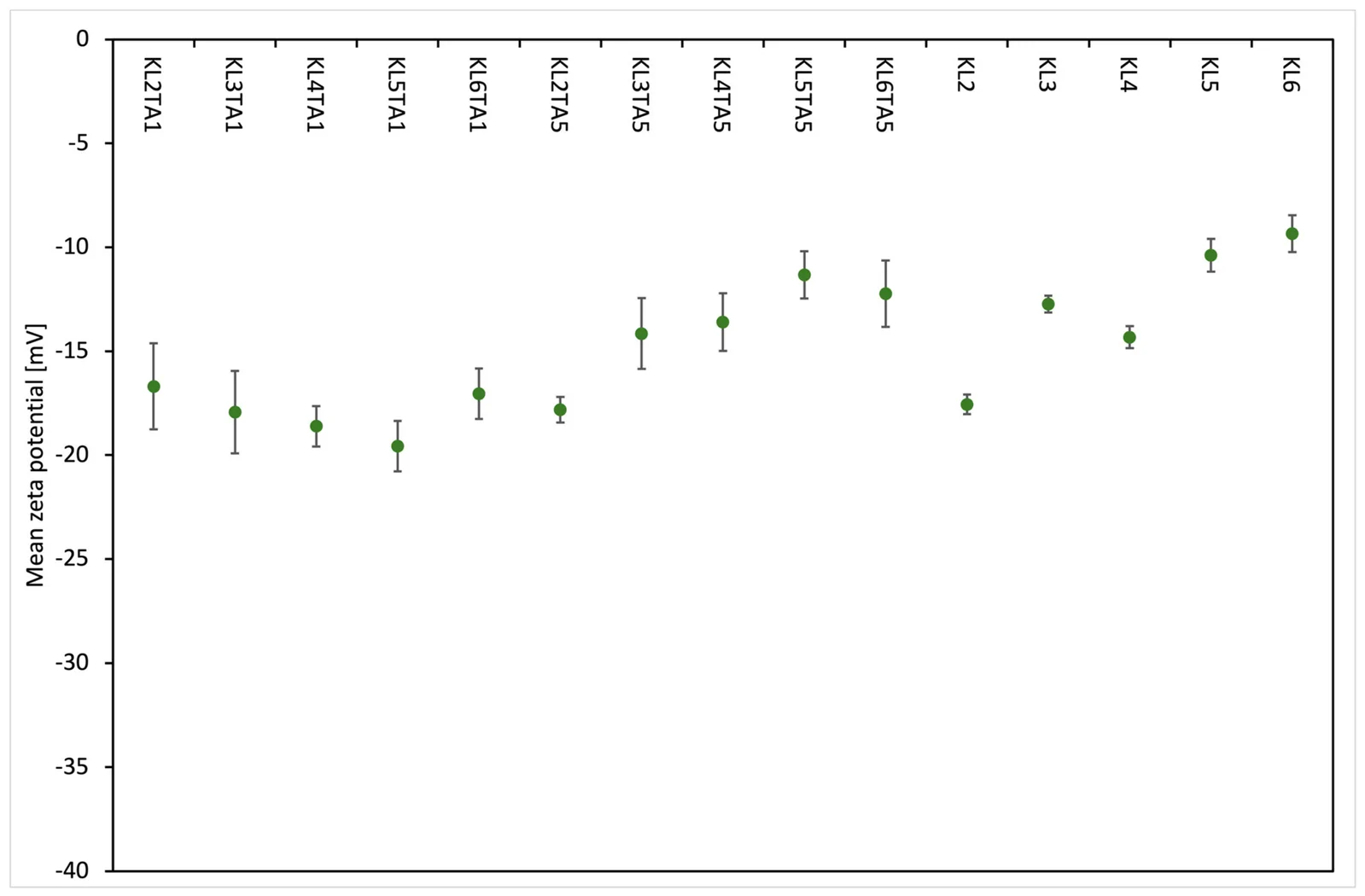
Zeta potential of the prepared 5% *w*/*w* retinyl palmitate-loaded nanoemulsions containing Kolliphor EL as surfactant and Triacetin as cosurfactant.

The zeta potential values obtained for the prepared nanoemulsions were negative for all formulations. In the formulations containing TW, the zeta potential ranged from −32.14 ± 2.68 mV (TW2TA5) to −20.96 ± 0.62 mV (TW4), whereas the formulations containing KL exhibited values between −19.57 ± 1.21 mV (KL5TA1) and −9.35 ± 0.88 mV (KL6) (Figure 4 and Figure 5). In the literature, favorable physicochemical stability and promising properties for developed anionic emulsions in, e.g., ophthalmic use are also demonstrated [47].

#### 2.2.3. Characterization of Nanoemulsion Morphology by TEM

**Figure 6:**
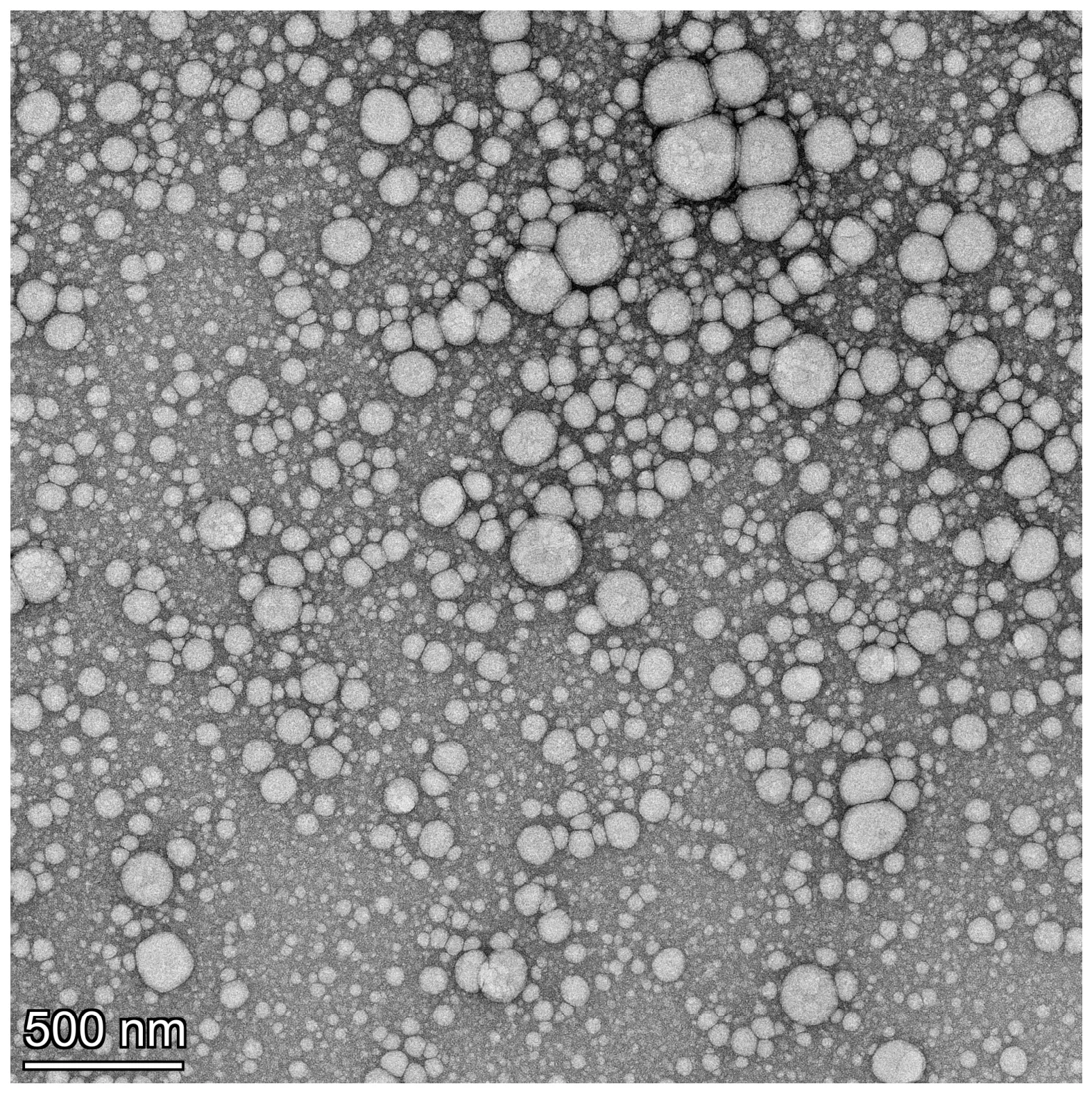
TEM image of 5% *w*/*w* retinyl palmitate-loaded nanoemulsion.

When analyzing the morphology and size of the selected nanoemulsion with transmission electron microscopy (TEM), spherical droplets were observed in the dispersed phase of TW5TA5 against the dark background, as visible in Figure 6. The size of the droplets was confirmed with TEM and is in agreement with the data in Table 1.

#### 2.2.4. Stability and Drug Content of Selected Nanoemulsions

All manufactured nanoemulsions were stored for 28 days at room temperature (25 ± 0.5 °C) and under refrigerated conditions (6.0 ± 0.5 °C). After the storage period, all emulsions were evaluated for any signs of nanoemulsion destabilization such as coalescence, flocculation, creaming, sedimentation, or phase separation. No visible signs of destabilization were observed in the tested formulations. Table 1 presents the changes in mean droplet diameter, polydispersity index (PDI), and zeta potential for the nanoemulsions that exhibited the most favorable physicochemical parameters on day 0. The nanoemulsion containing 5% Tween 20 and 5% Triacetin demonstrated the most stable and optimal characteristics both immediately after preparation and after 28 days of storage under different temperature conditions. This formulation maintained a consistent droplet size, low PDI values, and comparatively higher absolute zeta potential values, indicating good physical stability over time. However, retinyl palmitate content right after manufacturing was below the required 90%.

### 2.3. Characterization of Optimized Nanoemulsion with 5% w/w Retinyl Palmitate

#### 2.3.1. Viscosity Analysis

Viscosity is an important parameter influencing the residence time of a formulation on the skin’s surface and, consequently, the bioavailability of the active substance during microneedling application. The viscosity of the obtained nanoemulsion, measured at a shear rate of 1875 s^−1^ at 25.0 ± 1.0 °C, was found to be 1.77 ± 0.04 mPa·s, and decreased to 1.43 ± 0.02 mPa·s at 36.0 ± 1.0 °C. In contrast to oleogels, nanoemulsions are characterized by relatively low viscosity. This lower consistency may potentially provide an advantage by facilitating improved penetration and absorption of the active substance into the skin [48]. In clinical practice, low-viscosity fluids are commonly combined with microneedling, as formulations designed for standard mesotherapy are often used.

#### 2.3.2. pH

The acidic pH of the skin plays an important role in maintaining the proper activity of enzymes responsible for skin integrity and barrier function, while also contributing to antimicrobial protection [49]. The mean pH value of the selected nanoemulsion containing 5% Tween 20 and 5% Triacetin was 4.84 ± 0.04, which is within the physiologically acceptable range for topical formulations. According to the study by Kilic et al., an emulsion with a pH of approximately 4 was shown to improve the epidermal barrier in elderly patients [50]. These findings suggest that the developed nanoemulsion may support skin barrier function while remaining compatible with the natural skin environment.

### 2.4. In Vitro Cytotoxicity Determination of 5% w/w Retinyl Palmitate-Loaded Oleogel and Nanoemulsion in Comparison with Placebo

**Figure 7:**
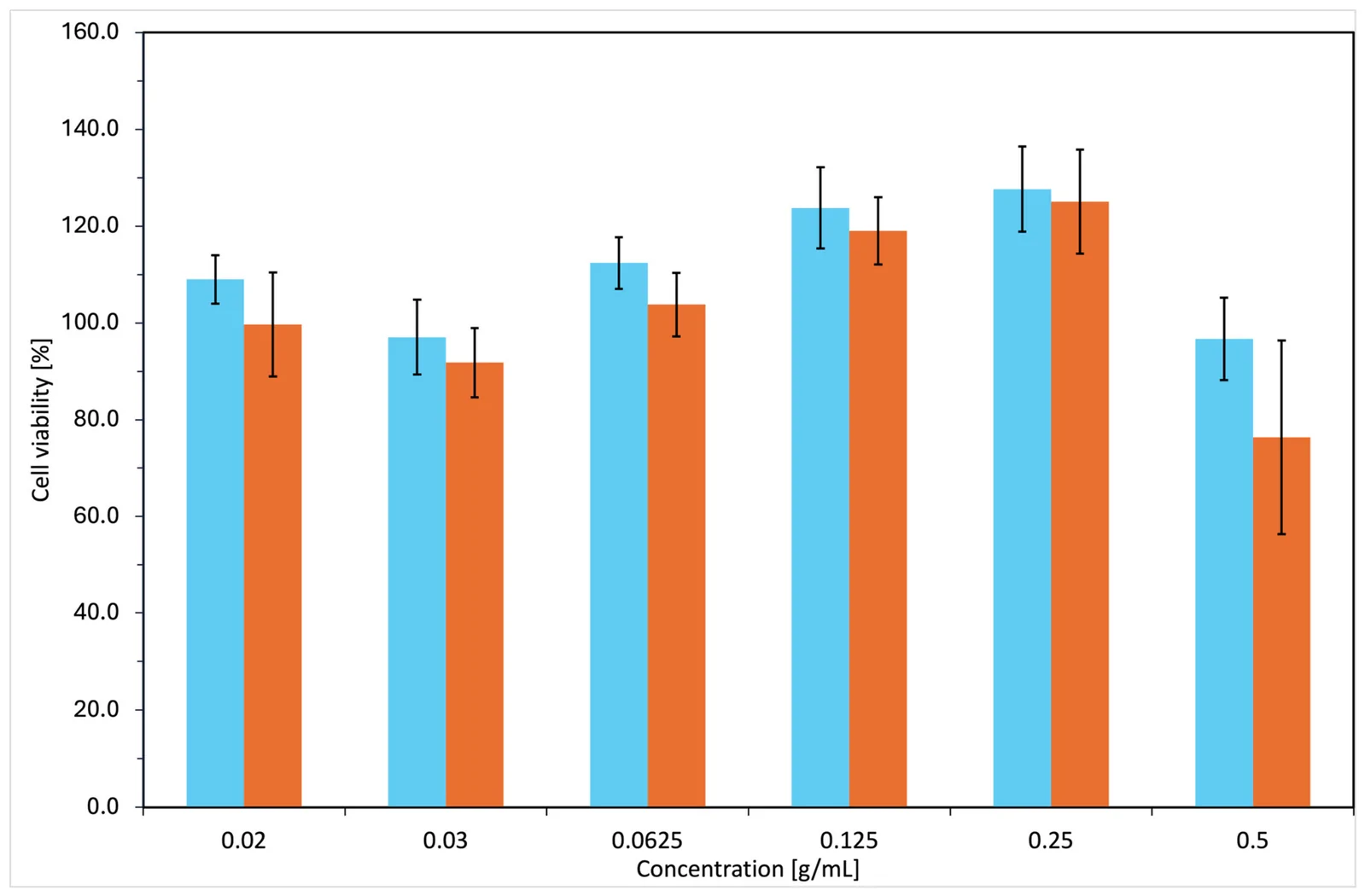
In vitro cytotoxicity determination of 5% *w*/*w* retinyl palmitate-loaded oleogel (orange bar) in comparison with placebo (blue bar).

As has been demonstrated in Figure 7, 5% *w*/*w* retinyl palmitate-loaded oleogel demonstrated low cytotoxicity, with cell viability remaining above 70% and placebo being slightly better tolerated. The obtained data is consistent with the publication by Oh et al. [51] where chitosan-coated nanocapsules were prepared to encapsulate retinyl palmitate. Such capsules, loaded with approximately 5% wt.% of retinyl palmitate, did not exhibit any cytotoxic effects on dermal fibroblasts.

### 2.5. Clinical Evaluation

An evaluation of the safety profile, tolerability, and effectiveness of the combination of 5% retinyl palmitate-loaded oleogel with a series of microneedling procedures was published in a study by Tobiasz et al., where good tolerability, safety profile and patients’ satisfaction with the treatment were described in patients with acne scars [52]. The 5% retinyl palmitate-loaded nanoemulsion has not been tested yet due to the necessary active substance content optimization process. Promising results observed in procedures with oleogel implicate the need for further studies to obtain a stable retinyl palmitate-loaded nanoemulsion.

### 2.6. Limitations of the Study

Testing only a high-pressure homogenization method was the main limitation of the study.

Both high- and low-energy nanoemulsion manufacturing methods exist, but low-energy methods seem to be less efficient and usually involve the use of greater amounts of surfactants. The main focus of the study was the use of minimal amounts of surfactants or any other stabilizing agents, due to the application of formulations on an interrupted skin barrier. Another issue that would appear in nanoemulsions manufactured with low-energy methods is inferior stability [25,53]. Therefore, further studies should include modifications to high-energy methods, which would minimize the loss of the active substance during high-pressure homogenization, e.g., the use of efficient cooling methods during high-pressure homogenization, dead volume reduction, and formulation overage [54].

Another limitation was the lack of in vitro permeation and release studies for the developed formulations. However, the oleogel formulation was subsequently evaluated in a preliminary clinical study involving patients with acne scars, where favorable tolerability and safety profiles were demonstrated [52].

## 3. Materials and Methods

### 3.1. Materials

Retinyl palmitate oil-based solution (European Pharmacopoeia 11.0.) was purchased from Caesar & Loretz GmbH—Caelo (Hilden, Germany); oleogel (combination of hypoallergenic liquid paraffin with 5% *w*/*w* high-pressure polyethylene) from Actifarm (Warsaw, Poland); surfactants Kolliphor EL (polyoxyl-35 castor oil, KL), Tween 20 (polyoxyethylene sorbitan monolaurate, TW), and cosurfactant Triacetin (glycerol triacetate, TA) from Sigma-Aldrich Chemical Company (St. Louis, MO, USA). For high-performance liquid chromatography (HPLC), HPLC-grade methanol and n-hexane absolute were purchased from Sigma-Aldrich Chemical Company (St. Louis, MO, USA). All other chemicals and solvents used were of analytical grade.

### 3.2. Preparation of the 5% w/w Retinyl Palmitate-Loaded Oleogel

Retinyl palmitate-loaded oleogel was prepared under aseptic conditions in accordance with guidelines for semisolid formulations. Firstly, the oleogel base was sterilized in a drying oven, Pol-Eko (Wodzisław Śląski, Poland), according to parameters specified in the Polish Pharmacopeia (180 °C, 30 min.) and in compliance with the manufacturer’s recommendations [27]. All necessary utensils resistant to heat were sterilized; the rest of the utensils were cleaned with 70% (*v*/*v*) ethanol, while some of the required utensils were already provided in a sterile form. A laminar airflow cabinet was appropriately cleaned, disinfected and exposed to a germicidal UV lamp. The retinyl palmitate solution in peanut oil was filtered through MCE 0.22 µm sterile filter, Filtrakon (Łaziska Górne, Poland). Subsequently, a 5% *w*/*w* retinyl palmitate-loaded oleogel formulation was prepared aseptically in a laminar flow cabinet SCANLAF Mars Pro Cytosafe Class 2, Innoguard (Höör, Sweden). Two preparation techniques were employed. In the first method, the preparations were prepared using an Unguator^®^ e/s, Gako Deutschland GmbH (Scheßlitz, Germany), with a mixing speed at level 1 of 810 rpm for 30 s. In the second method, the formulations were obtained using a mortar and pestle, with gentle mixing until a homogeneous system was obtained.

### 3.3. Physicochemical Characterization of the Obtained 5% w/w Retinyl Palmitate-Loaded Oleogel Preparations

In order to evaluate the prepared 5% *w*/*w* retinyl palmitate-loaded oleogel in terms of physicochemical properties, stability, and cytotoxicity multiple tests described in the following sections were performed.

#### 3.3.1. Viscosity Measurement

Samples of 5% *w*/*w* retinyl palmitate-loaded oleogel were analyzed at two different temperatures, 25.0 ± 1.0 °C and 36.0 ± 1.0 °C. The equipment used for this procedure was a Brookfield RV DV III+ Digital Rheometer, Brookfield Engineering Laboratories, Inc. (Middleboro, MA, USA) with a Brookfield CPE 51 Spindle and parameters such as speed rate 50 rpm and shear rate 192 s^−1^. Each sample was tested in triplicate, and the results are presented as the mean ± standard deviation.

#### 3.3.2. Spreadability Determination

The oleogels’ spreadability was evaluated using plexiglass plates and 150 g weights by placing 1.0 cm^3^ of 5% *w*/*w* retinyl palmitate-loaded oleogel at the center of a plexiglass plate (10 cm × 10 cm). The second identical plexiglass plate was placed on top, and the diameter of the spread was measured afterwards, after adding each of the 150 g weights until achieving 1200 g of load. Measurements were performed in triplicate at room temperature (25.0 °C). The spreadability was determined as spreading area diameter (mm^2^) depending on the upper loaded-plate mass [g]. The results are presented as the mean ± standard deviation.

#### 3.3.3. Drug Content Evaluation

To determine the content of retinyl palmitate in the obtained preparations, from each formulation, 3 weighted portions were prepared and diluted with n-hexane to a total volume of 10 mL. The resulting samples were put into the ultrasound bath for 20 min at 25.0 °C. Then, the samples were filtered through a PTFE 0.45 μm filter, Biosens (Warsaw, Poland). Drug content was assessed using high-performance liquid chromatography (HPLC), as described in Section 3.3.4. The results are presented as the mean ± standard deviation.

#### 3.3.4. High-Performance Liquid Chromatography Method (HPLC) for Retinyl Palmitate Determination

The retinyl palmitate content was assayed using an Agilent 1260 Infinity (Agilent Technologies, Inc., Santa Clara, CA, USA), equipped with a UV-DAD detector and Zorbax SB-C18 column 4.6 mm × 150 mm with 5 µm particles (Agilent Technologies, Inc., Santa Clara, CA, USA). As the mobile phase, methanol with water in a 98:2 *v*/*v* ratio was used. The injection volume was 10 µL, and the column temperature was maintained at 40 °C. Retinyl palmitate detection was recorded at the wavelength of 325 nm with a retention time of 17.15 min. Retinyl palmitate content was calculated using a calibration curve created in the concentration range of 47.652–4765.200 µg/mL with a mean correlation coefficient of R^2^ = 0.9997.

#### 3.3.5. Short-Term Stability Evaluation

To monitor chemical stability, the prepared samples were stored at 6 ± 0.5 °C for 28 days. The preparations were evaluated for drug content, and signs of destabilization such as a change in consistency or color at 4 time points: after manufacturing, after 10 days, after 20 days, and after 28 days. The results are presented as the mean ± standard deviation.

#### 3.3.6. In Vitro Cytotoxicity Determination of 5% *w*/*w* Retinyl Palmitate Loaded in Comparison with Placebo

The L929 murine fibroblast cell line (ATCC CCL) was used to evaluate the cytotoxicity of the tested samples. The cells were cultured in Dulbecco’s modified eagle’s medium (DMEM, Biowest, Riverside, MO, USA) supplemented with 1% (*v*/*v*) penicillin/streptomycin mixture (Biowest, Riverside, MO, USA), 1% (*v*/*v*) amphotericin B (Biowest, Riverside, MO, USA), and 10% (*v*/*v*) fetal bovine serum FBS (Biowest, Riverside, MO, USA). A total of 1 × 10^4^ cells per well were seeded in 96-well plates (VWR, Radnor, PA, USA) and incubated at 37 °C, 5% CO_2_ (Binder C 150 UL CO_2_ incubator, Tuttlingen, Germany) for 24 h. Simultaneously, the samples of oleogel formulations and the corresponding placebo controls were prepared as follows. First, 1 g of the oleogel was added to the wells of a 6-well plate (VWR, Radnor, PA, USA) in three repetitions. Next, 2 mL of the culture medium was added, and the plates were shaken at 800 rpm (Plate Shaker-Thermostat PST-60HL-4 (Biosan, Riga, Latvia)) at 8 °C for 18 h. Therefore, the concentration of applied oleogel was expressed as g of oleogel/mL medium. The medium was then collected, and the sample was centrifuged twice at 1000 rpm. The supernatant was collected, geometrically diluted in the medium, and further used for cell treatment. After incubation, the medium above the cells was gently removed, and 100 µL of the test compounds or medium alone (growth control) was added. The plates were incubated for 24 h. Next, the medium was replaced with 100 µL of the solution of 0.5 mg/mL MTT ((3-(4,5-dimethyl-2-thiazolyl)-2,5-diphenyl-2H-tetrazolium bromide) (Sigma Aldrich, Schnelldorf, Germany) in DMEM and the setting was incubated for 3 h under the same conditions. The dye was gently removed, and 100 µL of DMSO (dimethyl sulfoxide) was added to dissolve crystals. A total of 50 µL of the color solution was transferred to a fresh plate, and absorbance was measured at 570 and 630 nm using a spectrophotometer (Multiscan^®^ GO, Thermo Scientific, Waltham, MA, USA). The values at 630 nm were subtracted from the values at 570, and the viability of the cells treated with the tested formulations was calculated using the formula:

$$Cells\;Viability\left[\%\right]=\left(\frac{AbS}{AbC}\right)\times 100\%$$

where AbS is the mean absorbance value of the treated samples, and AbC is the mean values of the growth control.

The experiment was conducted as a single independent experiment. For the oleogel samples, three repetitions were performed from each of three samples. Outliers were defined as values more than 2× the standard deviation from the mean. The results are presented as the mean ± standard deviation.

### 3.4. Optimization of 5% w/w Retinyl Palmitate-Loaded Nanoemulsion

To select the optimal composition, various concentrations of surfactants (ranging from 2 to 6%) TW and KL, in combination with different concentrations of TA (1% and 5%) as a cosurfactant, were tested. The applied compositions are presented in Table 2. An oil solution of retinyl palmitate was mixed with a proper surfactant (TW/KL) and cosurfactant (TA) for 1 h using a magnetic stirrer, IKA Industrie (Königswinter, Germany), at 300 rpm. After adding the deionized water, the mixture was again mixed for 1 h using a magnetic stirrer, IKA Industrie (Königswinter, Germany), at 300 rpm. The homogenization parameters, including pressure and number of cycles, were adopted from a previously optimized study [55]. To obtain a nanoemulsion, samples were homogenized using a PandaPlus 2000 high-pressure homogenizer (GEA Mechanical Equipment, Parma, Italy), 3 cycles at a pressure of 1000 bar. The measurement of nanoemulsion formulation temperature after 3 homogenization cycles did not exceed 35 °C. To obtain a sterile formulation, the nanoemulsion was filtered in a laminar airflow cabinet, using properly sterilized utensils and pyrogen-free MCE membrane filters with a pore size of 0.22 μm (Filtrakon, Laziska Gorne, Poland).

#### 3.4.1. Measurement of Particle Size (PS) and Polydispersity Index (PDI)

The mean particle size (PS), polydispersity index (PDI), and particle size distribution by intensity of the tested nanoemulsions were measured by the dynamic light-scattering (DLS) method using a Zetasizer Nano ZS ZEN3600 (Malvern Instruments Ltd., Worcestershire, UK), fitted with a 4 mW He-Ne laser operating at 633 nm. Before performing measurements, each nanoemulsion was diluted using deionized water to 1:50 *v*/*v* to avoid multiple scattering effects. The temperature of measurements was 25.0 ± 0.5 °C. The results are expressed as the mean value of the size (intensity mean) and standard deviation of three independent measurements. The results are presented as the mean ± standard deviation.

#### 3.4.2. Measurement of Zeta Potential (ZP)

Zeta potential was measured using the Zetasizer Nano ZS ZEN3600 (Malvern Instruments Ltd., Worcestershire, UK) using electrophoretic light-scattering methods. Before performing measurements each nanoemulsion was diluted using deionized water in a 1:50 *v*/*v* ratio. The temperature of measurements was 25.0 °C. The results are presented as the mean ± standard deviation.

#### 3.4.3. Surface Morphology—Transmission Electron Microscopy (TEM)

The inner phase droplet size, shape, and structure of the nanoemulsions were determined using a Talos F200i transmission electron microscope (Thermo Fisher Scientific, Waltham, MA, USA) operated at an accelerating voltage of 200 kV. A droplet of the 10-fold-diluted nanoemulsion dispersion was deposited onto a 200-mesh carbon-coated copper grid (AGS160, Agar Scientific, Rotherham, UK) and negatively stained with a 2% (*w*/*v*) aqueous uranyl acetate solution, and blotted to remove excess staining before air drying. TEM images were acquired using a Ceta 16M CMOS camera (Thermo Fisher Scientific, Waltham, MA, USA) controlled with Velox software (version 3.13, Thermo Fisher Scientific, Waltham, MA, USA).

### 3.5. Physicochemical Properties of the Optimal 5% w/w Retinyl Palmitate-Loaded Nanoemulsion

In order to evaluate the prepared 5% *w*/*w* retinyl palmitate-loaded nanoemulsion in terms of physicochemical properties and stability, multiple tests as described in the following sections were performed.

#### 3.5.1. Viscosity

The viscosity of the samples (0.5 mL) was determined in triplicate at 25 ± 1.0 °C and 36 ± 1.0 °C under a speed rate of 250 rpm and a shear rate of 1875 s^−1^ using a Brookfield RV DV III+ Digital Rheometer, Brookfield Engineering Laboratories, Inc. (Middleboro, MA, USA), equipped with a Brookfield CPE 40 Spindle. The results are presented as the mean ± standard deviation.

#### 3.5.2. pH Measurement

The pH value of the selected 5% *w*/*w* retinyl palmitate-loaded nanoemulsion was performed in triplicate potentiometrically at 25.0 °C using a CPC-511 pH-meter, Elmetron (Zabrze, Poland) with an electrode InLab^®^ Flex-Micro, Mettler Toledo (Warsaw, Poland). The results are presented as the mean ± standard deviation.

#### 3.5.3. Drug Content Determination

For the quantitative determination of the retinyl palmitate content in the obtained preparations, 3 weighted portions of the obtained formulations were prepared and diluted with n-hexane to a total volume of 10 mL. The resulting samples were put into the ultrasound bath for 20 min at 25.0 °C. Then, the samples were filtered through a PTFE 0.45 μm filter, Biosens (Warsaw, Poland). Drug content was assessed via the HPLC method described in Section 3.3.4. The relevant HPLC-related data and sample spectra for the pure drug and drug-loaded formulations are available in Tables S1 and S2 in the Supplementary Materials.

#### 3.5.4. Short-Term Stability Determination

To evaluate the short-term stability of the prepared nanoemulsions, samples were stored in amber glass vials at room temperature (25.0 ± 0.5 °C) and in a refrigerator (6.0 ± 0.5 °C) for 28 days. The samples were evaluated for any indications of phase separation, drug precipitation, changes in droplet size, PDI values, particle surface charge, and drug content at 4 time points: after homogenization, after 10 days, after 20 days, and after 28 days. The results are presented as the mean ± standard deviation.

## 4. Conclusions

The developed 5% *w*/*w* retinyl palmitate-loaded oleogel and nanoemulsion represent promising formulations for potential combination with microneedling procedures in patients with acne scars. Both systems demonstrated favorable physicochemical characteristics, including appropriate viscosity, spreadability, particle size distribution, and pH values suitable for topical application on compromised skin.

The oleogel formulation fulfilled pharmacopeial requirements regarding active substance content and maintained satisfactory stability during storage. In contrast, despite achieving desirable nanoemulsion parameters such as low particle size, narrow polydispersity index, and acceptable zeta potential values, sufficient chemical stability of retinyl palmitate during manufacturing and storage was not fully achieved. The observed active substance content reduction was likely associated with losses during manufacturing and the high sensitivity of retinyl palmitate to processing conditions during high-pressure homogenization.

The development of stable retinyl palmitate-loaded nanoemulsions containing low concentrations of surfactants remains challenging yet clinically relevant due to their potential use in combination with microneedling procedures on the disrupted skin barrier. Further optimization of manufacturing conditions is therefore necessary to improve retinoid stability while maintaining favorable safety and application properties.

## Figures and Tables

**Table 1:** Parameter comparison of nanoemulsions which exceeded the most optimal parameters.

Formulation Code	Mean Droplet Diameter [nm]			Polydispersity Index			Zeta Potential [mV]		
	Day 0	Day 28 6 ± 0.5 °C	Day 28 25 ± 1 °C	Day 0	Day 28 6 ± 0.5 °C	Day 28 25 ± 1 °C	Day 0	Day 28 6 ± 0.5 °C	Day 28 25 ± 1 °C
	M ± SD	M ± SD	M ± SD	M ± SD	M ± SD	M ± SD	M ± SD	M ± SD	M ± SD
TW5TA5	130.32 ± 1.16	130.59 ± 1.16	129.34 ± 1.43	0.163 ± 0.012	0.160 ± 0.014	0.148 ± 0.011	−22.04 ± 1.28	−19.14 ± 1.19	−14.17 ± 0.34
TW6TA5	127.24 ± 0.61	127.74 ± 1.59	126.57 ± 1.38	0.168 ± 0.010	0.163 ± 0.012	0.163 ± 0.010	−21.31 ± 0.48	−15.24 ± 0.63	−12.81 ± 0.61
KL5TA5	131.44 ± 0.93	133.34 ± 1.71	135.67 ± 5.76	0.154 ± 0.015	0.157 ± 0.014	0.178 ± 0.038	−11.33 ± 1.13	−6.23 ± 1.35	−3.49 ± 0.53
KL6TA5	128.62 ± 1.52	130.08 ± 1.84	128.69 ± 1.10	0.146 ± 0.013	0.157 ± 0.024	0.147 ± 0.015	−12.23 ± 1.60	−4.88 ± 0.40	−3.13 ± 0.24

The selected nanoemulsion is highlighted in yellow.

**Table 2:** Composition of 5% *w*/*w* retinyl palmitate-loaded nanoemulsions.

Formulation Symbol	Compositions [g]				
		Surfactants		Cosurfactant	
	Retinyl Palmitate Oil Solution * [g]	TW [g]	KL [g]	TA [g]	Water [g]
TW2TA1	1.8	0.4	0	0.2	17.6
TW3TA1	1.8	0.6	0	0.2	17.4
TW4TA1	1.8	0.8	0	0.2	17.2
TW5TA1	1.8	1.0	0	0.2	17.0
TW6TA1	1.8	1.2	0	0.2	16.8
TW2TA5	1.8	0.4	0	1.0	16.8
TW3TA5	1.8	0.6	0	1.0	16.6
TW4TA5	1.8	0.8	0	1.0	16.4
TW5TA5	1.8	1.0	0	1.0	16.2
TW6TA5	1.8	1.2	0	1.0	16.0
KL2TA1	1.8	0	0.4	0.2	17.6
KL3TA1	1.8	0	0.6	0.2	17.4
KL4TA1	1.8	0	0.8	0.2	17.2
KL5TA1	1.8	0	1.0	0.2	17.0
KL6TA1	1.8	0	1.2	0.2	16.8
KL2TA5	1.8	0	0.4	1.0	16.8
KL3TA5	1.8	0	0.6	1.0	16.6
KL4TA5	1.8	0	0.8	1.0	16.4
KL5TA5	1.8	0	1.0	1.0	16.2
KL6TA5	1.8	0	1.2	1.0	16.0
TW2	1.8	0.4	0	0	17.8
TW3	1.8	0.6	0	0	17.6
TW4	1.8	0.8	0	0	17.4
TW5	1.8	1.0	0	0	17.2
TW6	1.8	1.2	0	0	17.0
KL2	1.8	0	0.4	0	17.8
KL3	1.8	0	0.6	0	17.6
KL4	1.8	0	0.8	0	17.4
KL5	1.8	0	1.0	0	17.2
KL6	1.8	0	1.2	0	17.0

* Vitamin A solution 1 MIU/g in peanut oil; TW—Tween 20, KL—Kolliphor EL, TA—Triacetin.

## Data Availability

The original contributions presented in this study are included in the article/Supplementary Material. Further inquiries can be directed to the corresponding authors.

## Supplementary Materials

The following supporting information can be downloaded at: https://www.mdpi.com/article/10.3390/ph19081247/s1, Table S1: HPLC analyzed samples – data.; Table S2: Chromatograms of formulations.

## Abbreviations

The following abbreviations are used in this manuscript:

ATCC	American Type Culture Collection
ECM	Extracellular Matrix
DMEM	Dulbecco’s modified eagle’s medium
DMSO	Dimethyl sulfoxide
HPH	High-pressure homogenization
HPLC	High-performance liquid chromatography
KL	Kolliphor EL^®^
MCE	Mixed Cellulose Ester
MTT	3-(4,5-dimethyl-2-thiazolyl)-2,5-diphenyl-2H-tetrazolium bromide
o/w	Oil-In-Water
PDI	Polydispersity Index
PRP	Platelet-rich plasma
PS	Particle Size
PTFE	Polytetrafluoroethylene
RAR	Retinoic Acid Receptor
RXR	Retinoid X Receptor
TA	Triacetin
TEM	Transmission Electron Microscopy
TW	Tween 20
UV	Ultraviolet
Vit. A	Vitamin A
w/o	Water-In-Oil
