# Supplementary material for: Development and Optimization of Retinyl Palmitate-Loaded Oleogel and Nanoemulsion Designed with Potential for Microneedling Treatment
Source: Pharmaceuticals (Basel). 2026 Aug 8;19(8):1247. doi: 10.3390/ph19081247 (PMC13516896; doi:10.3390/ph19081247)
Supplement: Supplementary file 1 [file pharmaceuticals-19-01247-s001.zip › pharmaceuticals-4367073-supplementary.pdf]

Table S1. HPLC analyzed samples – data.

Sample name	Amount of sample weighted [g]	Measured concentration in sample [ug/ml]	% declared API content
**5% w/w Retinyl Palmitate loaded Oleogel I - Unguator**	0.736	3248.611	89.04
**5% w/w Retinyl Palmitate loaded Oleogel II - Unguator**	0.717	3120.843	87.80
**5% w/w Retinyl Palmitate loaded Oleogel III - Unguator**	0.717	3143.346	88.44
**5% w/w Retinyl Palmitate loaded Oleogel I – Mortar Day 0**	0.539	2703.512	99.57
**5% w/w Retinyl Palmitate loaded Oleogel II – Mortar Day 0**	0.580	2949.053	100.95
**5% w/w Retinyl Palmitate loaded Oleogel III– Mortar Day 0**	0.519	2622.327	100.30
**5% w/w Retinyl Palmitate loaded Oleogel I – Mortar Day 28**	0.468	2196.907	93.19
**5% w/w Retinyl Palmitate loaded Oleogel II – Mortar Day 28**	0.448	2162.225	95.81
**5% w/w Retinyl Palmitate loaded Oleogel III – Mortar Day 28**	0.631	3047.980	95.89
**TW5TA5 I**	0.190	702.492	74.02
**TW5TA5 II**	0.200	635.884	63.65
**TW5TA5 III**	0.190	703.492	74.13

*HPLC – High-Performance Liquid Chromatography; API – Active Pharmaceutical Ingredient

Table S2. Chromatograms of formulations.

Analyzed formulation	Chromatogram
Retinyl palmitate standard	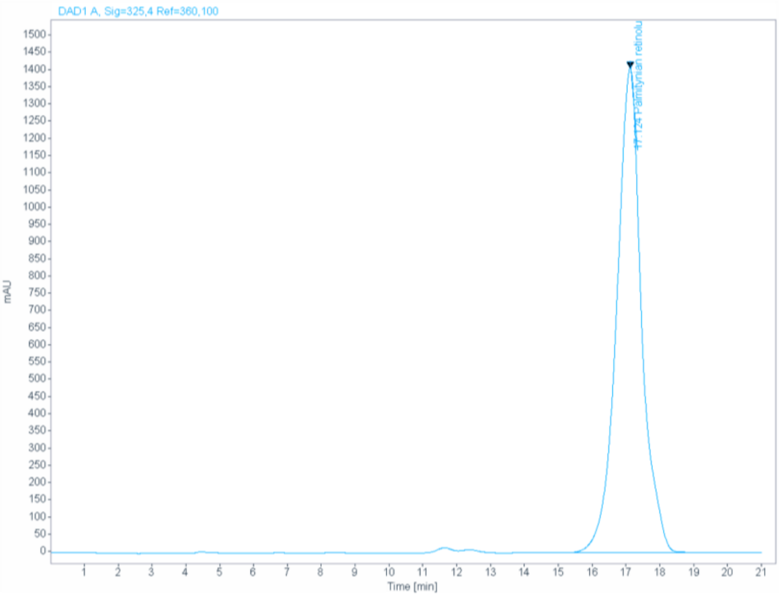
5% w/w Retinyl Palmitate Loaded Oleogel Day 0 Manufactured in Unguator	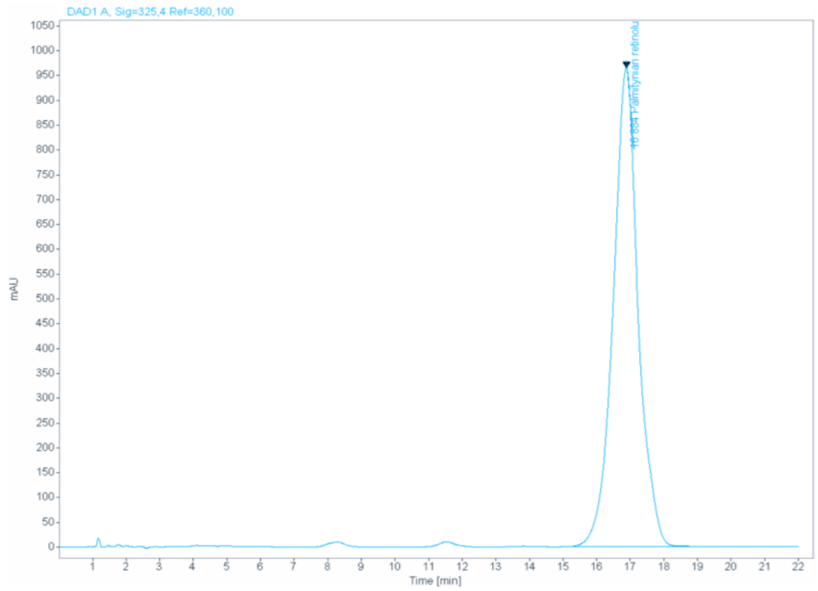

5% w/w Retinyl Palmitate Loaded Oleogel Day 0 Manufactured in Mortar	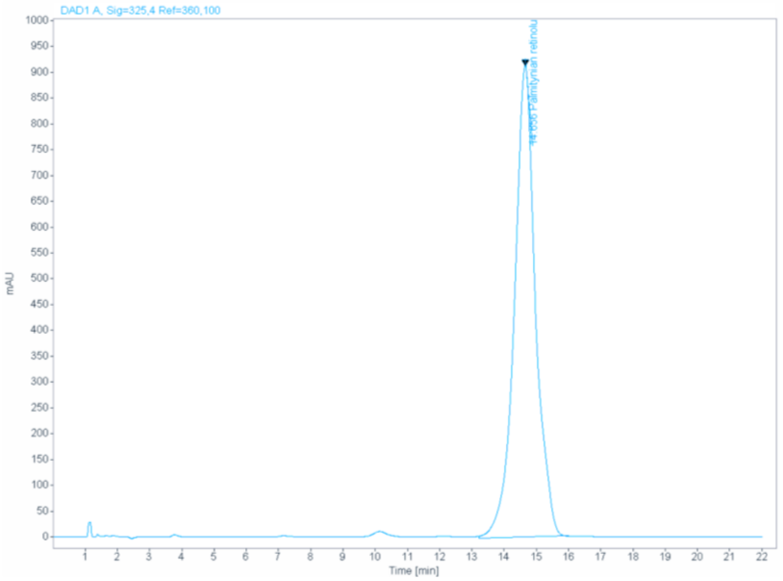
5% w/w Retinyl Palmitate Loaded Oleogel Day 28 Manufactured in Mortar	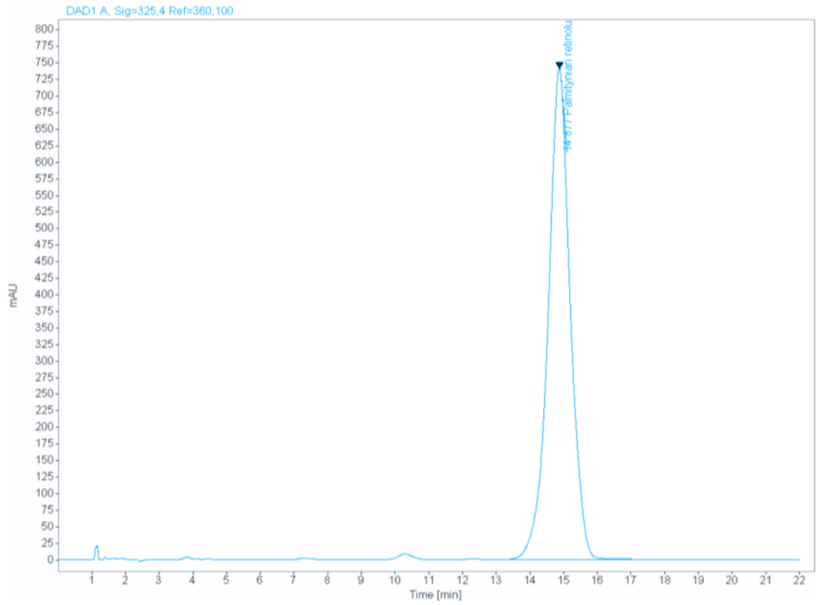

5% w/w Retinyl Palmitate Loaded Nanoemulsion TW5TA5 Day 0	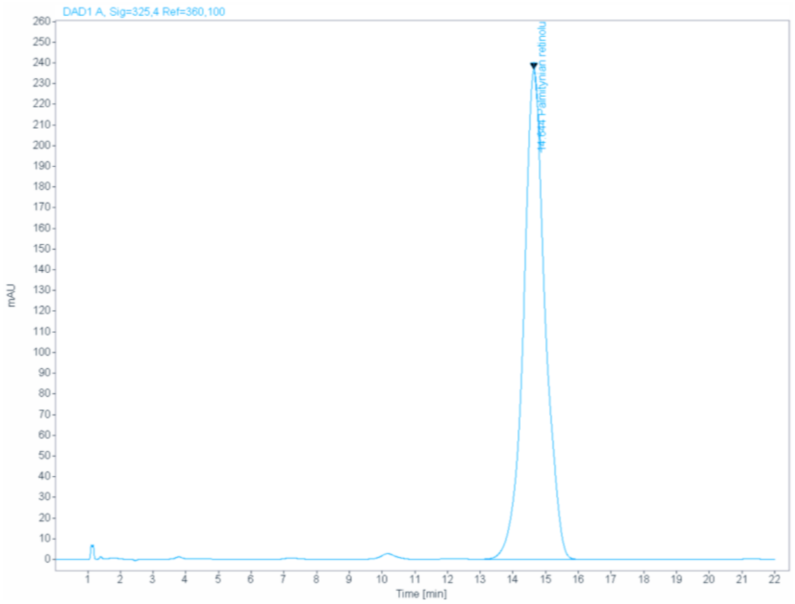
